# How do we choose the best donor for T-cell-replete, HLA-haploidentical transplantation?

**DOI:** 10.1186/s13045-016-0265-2

**Published:** 2016-04-12

**Authors:** Ying-Jun Chang, Leo Luznik, Ephraim J. Fuchs, Xiao-Jun Huang

**Affiliations:** Peking University People’s Hospital and Peking University Institute of Hematology, Beijing Key Laboratory of Hematopoietic Stem Cell Transplantation, No. 11 South Street of Xizhimen, Beijing, Xicheng District 100044 China; Sidney Kimmel Comprehensive Cancer Center, Johns Hopkins University School of Medicine, 1650 Orleans Street, Baltimore, MD 21287 USA; Peking-Tsinghua Center for Life Sciences, Beijing, 100871 China

**Keywords:** Unmanipulated haploidentical stem cell transplantation, Donor, Donor-specific anti-human leukocyte antigen antibody, Natural killer alloreactivity, Age, Non-inherited maternal antigen mismatch

## Abstract

In haploidentical stem cell transplantations (haplo-SCT), nearly all patients have more than one donor. A key issue in the haplo-SCT setting is the search for the best donor, because donor selection can significantly impact the incidences of acute and chronic graft-versus-host disease, transplant-related mortality, and relapse, in addition to overall survival. In this review, we focused on factors associated with transplant outcomes following unmanipulated haplo-SCT with anti-thymocyte globulin (ATG) or after T-cell-replete haplo-SCT with post-transplantation cyclophosphamide (PT/Cy). We summarized the effects of the primary factors, including donor-specific antibodies against human leukocyte antigens (HLA); donor age and gender; killer immunoglobulin-like receptor-ligand mismatches; and non-inherited maternal antigen mismatches. We also offered some expert recommendations and proposed an algorithm for selecting donors for unmanipulated haplo-SCT with ATG and for T-cell-replete haplo-SCT with PT/Cy.

## Background

Allogeneic hematopoietic cell transplantation (SCT) remains the only curative therapy for a majority of malignant hematologic diseases [1–7]. Currently, for patients that require transplantation, but have no related or unrelated donors with matching human leukocyte antigen (HLA), the HLA-haploidentical SCT (haplo-SCT) approach is an attractive option. Haplo-SCT is widely available, and immediate access is possible with donor-derived cellular therapies [1, 5, 6, 8–39]. Many haplo-SCT protocols have been successfully established, with promising clinical outcomes, due to improved understanding of the mechanisms underlying the HLA barriers and how to cross them [1, 8, 16, 24, 26, 40–44]. Approaches for T-cell depletion (TCD) transplantation, including “megadose” CD34-selection and TCRαβ depletion, are designed to preserve γδ T cells, natural killer (NK) cells, and antigen-presenting cells. Alternatively, several approaches are also available for T-cell-replete (TCR) transplantation, including anti-thymocyte globulin (ATG)-based protocols or post-transplantation cyclophosphamide (PT/Cy). Haplo-SCT has become easier to perform than in the past, thanks to the shift from T-depleted grafts to grafts composed of unmanipulated marrow and/or peripheral blood stem cells [5, 6, 10, 19, 21, 30, 31, 34, 45, 46]. In particular, the “GIAC” protocol for haplo-SCT with ATG is a procedure that includes “G”: treating donors with granulocyte colony-stimulating factor (G-CSF), to induce donor immune tolerance [47–51]; “I”: intensified immunologic suppression in the recipient, to promote engraftment and prevent GVHD; “A”: anti-thymocyte globulin administration for prophylaxis of graft-versus-host disease (GVHD) and graft rejection; and “C”: combining G-CSF-primed bone marrow cells (G-BM) and G-CSF-mobilized peripheral blood stem cells (G-PB) harvested from donors to provide a pooled source of stem cells for grafting [1, 52–55]. Results from two additional trials from Lee et al. in Korea [56] and Di Bartolomeo et al. [57] from Italy largely reproduced the data from us; the effectiveness of the “GIAC” protocol was verified by these two external trials. As a result of these trends, the number of treatments that applied unmanipulated haplo-SCT with ATG or PT/Cy [1, 36, 58, 59] has increased significantly every year in China, the USA, and Europe (Table 1) [36–38, 60–66]. Some experienced centers, such as Peking University and Johns Hopkins University, have adopted haplo-SCT as the main source of alternative donors, based on outcomes that compared favorably with HLA-matched sibling or unrelated donor transplantations (MUDT) [17, 22, 62, 67]. With the advancement of haplo-SCT, particularly unmanipulated haplo-SCT, parents, children, siblings, and second-degree relatives, have all become potential donor candidates. Because most patients have more than one potential haplo donor, this raises an important question [68]: Who is the best donor for TCR haplo-SCT? Indeed, haplo donor selection may have a significant impact on the incidence of acute and chronic GVHD, transplant-related mortality (TRM), and relapse, in addition to overall survival (OS) [9–13, 19, 34, 36, 69–72]. In this review, we discuss the effects of HLA mismatching on transplant outcomes in patients treated with unmanipulated haplo-SCT with ATG [1, 52–55] or with PT/Cy [1, 36, 58, 59]. We also summarize donor-related variables that are associated with clinical outcomes, and we provide a rationale for using a personalized algorithm for donor selection in unmanipulated haplo-SCT with ATG [1, 52–55] or PT/Cy [1, 36, 58, 59].

**Table 1:** Recent informative trials and results of T-cell-replete haploidentical stem cell transplantation

Reference, year, ref, and median age	Patients (No.)	Diagnosis	Graft	ANC median (range)	PLT median (range)	GVHD		TRM	Relapse	LFS	OS
						Acute II–IV	Chronic				
Unmanipulated haplo-SCT with ATG											
Di Bartolomeo P, et al. [57] (37)	80	HM	G-BM	21 (12–38)	28 (14–185)	24 %	17 % at 2 yr	36 % at 3 yr	21 % at 1 yr	38 % at 3 yr	45 % at 3 yr
Wang Y, et al. [10] (25)	1210	HM	G-BM + G-PB	13 (8–49)	16 (5–100)	40 %	50 %	17 % at 3 yr	17 % at 3 yr	67 % at 3 yr	70 % at 3 yr
Luo Y, et al. [14] (25)	99	HM	G-PB	12 (8–24)	15 (6–53)	42.4 %	41.4 % at 2 yr	30.5 % at 5 yr	14.2 % at 5 yr	58.3 % at 5 yr	60.8 % at 5 yr
Gao L, et al. [66] (25.4)	26	SAA	G-PB + G-BM	13 (11–19)	13 (10–21)	8.0 %	40 %	15.4 % at 2 yr	NA	NA	84.6 % at 2 yr
Peccatori J, et al. [19] (48)	121	HM	G-PB	17 (11–61)	19 (7–154)	35 %	47 % at 2 yr	31 % at 3 yr	48 % at 3 yr	20 % at 3 yr	25 % at 3 yr
Shin SH, et al. [29] (48)	60	MDS	G-PB	12 (8–23)	15 (6–132)	36.7 %	48.3 %	23.3 % at 2 yr	34.8 % at 2 yr	41.9 % at 2 yr	46.6 % at 2 yr
Yahng SA, et al. [120] (41)	80	AML	G-PB	11	10	47.5 %	45 %	12.2 % at 2 yr	26.6 % at 2 yr	61.1 % at 2 yr	66 % at 2 yr
Lin X, et al. [78] (23)	105	HM	G-PB	14 (10–25)	16 (9–38)	21.9 %	24.1 % at 2 yr	30.5 % at 3 yr	21.9 % at 3 yr	41.1 % at 3 yr	50.6 % at 3 yr
TCR haplo-SCT with PT/Cy											
Raiola AM, et al. [38] (45)	92	HM	SS-BM (92)	18 (11–32)	32 (5–83)	14 %	15 %	18 % at 1000 days	35 %	43 % at 4 yr	52 % at 4 yr
McCurdy SR, et al. [36] (55)	372	HM	SS-BM	90 %^a^	88 %^b^	32 % at 6 m	13 % at 2 yr	11 % at 1 yr	46 % at 3 yr	40 % at 3 yr	50 % at 3 yr
Bacigalupo A, et al. [37] (47)	148	HM	SS-BM	17 (13–32)	NA	18 %	20 % at 2 yr	14 % at 4 yr	27 % at 4 yr	NA	77 % for CR1 49 % for CR2 38 % for AD
Solomon SR, et al. [61] (46)	30	HM	G-PB	16 (NA)	25 (NA)	43 %	56 %	3 % at 2 yr	24 % at 2 yr	73 % at 2 yr	78 % at 2 yr
Cieri N, et al. [64] (55)	40	HM	G-PB	18 (13–45)	16 (9–100)	15 %	20 % at 1 yr	17 % at 1 yr	35 % at 1 yr	48 % at 1 yr	56 % at 1 yr
Esteves I, et al. [65] (17)	16	SAA	SS-BM (13) G-PB (3)	19 (16–29)	21 (20–29)	13 %	20 %	32.9 % at 1 yr	NA	NA	67.1 % at 1 yr
Ciurea SO, et al. [62] (NA)	104^c^	AML	SS-BM (85) G-PB (19)	90 %	88 %	16 %	30 % at 3 yr	14 % at 3 yr	44 % at 3 yr	NA	45 % at 3 yr
	88^d^	AML	SS-BM (77) G-PB (11)	93 %	88 %	19 %	34 % at 3 yr	9 % at 3 yr	58 % at 3 yr	NA	46 % at 3 yr
Kasamon YL, et al. [59] (61)	271	HM	SS-BM	88–93 %	84–89 %	33 % at 6 m	12 % at 1 yr	10 % at 1 yr	46 % at 3 yr	37 % at 3 yr	46 % at 3 yr

Published between 2013 and 2015

*Ref* reference, *Pts* patients, *No.* number, *ANC* absolute neutrophil count, *PLT* platelet, *GVHD* graft-versus-host disease, *TRM* transplant-related mortality, *LFS* leukemia-free survival, *OS* overall survival, *haplo-SCT* haploidentical stem cell transplantation, *ATG* anti-thymocyte globulin, *HM* hematological malignancies, *G-BM* granulocyte colony-stimulating factor (G-CSF)-primed bone marrow, *yr* year, *G-PB* G-CSF-mobilized peripheral blood stem cell grafts, *UCB* umbilical cord blood, *NA* not available, *AL* acute leukemia, *SAA* severe aplastic anemia, *MDS* myelodysplastic syndrome, *AML* acute myeloid leukemia, *PT/Cy* posttransplant cyclophosphamide, *SS-BM* steady-state bone marrow, *m* months, *AD* advanced disease

^a^Indicates the probability of neutrophil recovery by day 30

^b^Indicates the probability of platelet recovery ≥20,000/μL by day 60

^c^Indicates that patients received myeloablative conditioning regimens

^d^Indicates that patients received reduced intensity conditioning regimens

## Effects of the locus of HLA-mismatch on haplo-SCT outcomes

Before the year 2000, patients that received haplo-SCT had relatively poor transplant outcomes, due to the use of conditioning and GVHD prophylaxis regimens that were similar to those used for transplantations from HLA-matched donors [73, 74]. Anasetti et al. [73] found that the degree of recipient HLA incompatibility was associated with the incidence of severe acute GVHD. Indeed, survival decreased as the degree of HLA disparity increased. Szydlo et al. [74] showed that, among patients with early leukemia that received transplantations, the relative risks of treatment failure were 2.43 and 3.79, when related donors had one and two mismatched HLA loci, respectively, compared to when donors were HLA-matched siblings (the reference group). Among patients with more advanced leukemia that received transplantations, differences in treatment failure were less striking; the relative risks of treatment failure were 1.22 and 1.81, when related donors had one and two HLA antigen mismatches, respectively, compared to the reference group. These data suggested that clinical outcomes depend on the degree of HLA mismatching in the early stages of haplo-SCT, because of little knowledge on immune tolerance and less approaches to overcome the HLA barriers.

Over the last 10 years, haplo-SCT outcomes have substantially improved, due to the development of novel GVHD prophylaxis strategies, improved supportive care strategies, and application of new strategies for relapse prophylaxis and treatment (Table 1) [18, 19, 28, 36, 42, 62, 75–77]. In 2006, a group at the University of Peking reported that the cumulative incidences of acute and chronic GVHD were comparable among patients with one-, two-, or three-locus mismatches, when treated with unmanipulated haploidentical blood and marrow transplantations and an ATG conditioning regimen [52]. They also demonstrated that HLA mismatching had no effect on other transplantation outcomes, including relapse, leukemia-free survival (LFS), and OS [52]. These results were confirmed by researchers from Peking University [9–12] and other transplantation centers in China [14, 35, 78]. Kasamon et al. [59] confirmed the findings by Huang et al., when they showed that greater HLA disparity did not appear to worsen the overall outcome after non-myeloablative haploidentical bone marrow transplantation with a high-dose PT/Cy. In a prospective, multicenter phase I/II study on unmanipulated haplo-SCTs performed in five institutions in Japan, Ikegame et al. [77] reported that HLA disparity was not associated with GVHD, TRM, relapse, or survival. Similar results were observed in recent updated reports on haplo-SCT with TCD or TCR [34, 35, 62, 72].

In an unmanipulated haplo-SCT protocol, Huang et al. [79] found that the HLA-B + DR combination mismatch was an independent risk factor for grades II–III and III–IV acute GVHD in patients with chronic myeloid leukemia (CML). Huo et al. [80] demonstrated that the HLA-B mismatch was also an independent risk factor for acute GVHD and TRM in patients with hematological diseases. However, SCT is not a first-line treatment option for patients with CML; therefore, associations between specific HLA-locus mismatches and haplo-SCT outcomes should be prospectively investigated in other hematological diseases.

In summary, studies on unmanipulated haplo-SCT with ATG [1, 52–55] or with PT/Cy [1, 36, 58, 59] showed that HLA disparity did not impact outcome. However, for donor selection, some specific HLA-loci profiles remain to be explored. Nevertheless, more attention has been focused on how donor-related, non-HLA variables affect clinical outcomes.

## Donor selection based on non-HLA variables

Because the impact of HLA disparity on transplantation outcome has diminished, researchers are currently investigating the effects of other variables on survival after unmanipulated haplo-SCT with ATG [1, 52–55] or with PT/Cy [1, 36, 58, 59]. A number of donor-related factors should be considered in donor selection for haplo-SCT, including donor-specific anti-HLA antibodies (DSA) [12, 81, 82], donor age and gender [10, 83], ABO compatibility, natural killer (NK) cell alloreactivity [23, 84–86], and non-inherited maternal antigen (NIMA) mismatches (Table 2) [87–90].

**Table 2:** Variables considered for best donor selection in unmanipulated haplo-SCT with ATG or TCR haplo-SCT with PT/Cy

Variables		Unmanipulated haplo-SCT with ATG	Ref	TCR haplo-SCT with PT/Cy	Ref
DSA		DSA was associated with primary graft failure, including GR and PGF.	[12]	DSA was associated with an increased risk of graft failure.	[93]
Donor age		Young donor age (<30) was associated with decreased 2–4 acute GVHD, NRM, and superior survival.	[10]	No effect of donor age on clinical outcomes was found.	[59]
Donor gender		F-M (versus others) correlated with higher incidence of 2–4 acute GVHD.	[10, 14]	Male donors were associated with less NRM and better survival.	[36, 102]
NK alloreactivity		KIR-ligand mismatch was associated with inferior survival.	[23]	A survival benefit associated with donor-recipient mismatches of inhibitory KIR and KIR haplotype B donors.	[59]
NIMA mismatch		NIMA-mismatched was associated with a lower incidence of acute GVHD in unmanipulated haplo-SCT.	[10]	–	
Type of donor	Children	Children donors were associated with less acute GVHD than sibling donors.	[10]	–	
	Mather	Maternal donors were associated with more acute GVHD, chronic GVHD, and NRM.		–	
	Older sister	Older sister donors were inferior to father donors in NRM and survival.		–	
	Father	Father donors were associated with less acute GVHD, less NRM, and better survival than mother donors.		–	

*Haplo-SCT* haploidentical stem cell transplantation, *ATG* anti-thymocyte globulin, *TCR* T-cell replete, *PT/Cy* posttransplant cyclophosphamide, *Ref* reference, *DSA* donor-specific anti-human leukocyte antibody, *GR* graft rejection, *PGF* poor graft function, *NK* natural killer, *KIR* inhibitory killer cell immunoglobulin-like receptor, *NIMA* non-inherited maternal antigen, *GVHD* graft-versus-host disease, *NRM* non-relapse mortality, *F* female, *M* male

– indicates no data available

### DSA

The contribution of DSAs to the pathophysiology of graft failure (GF) has been confirmed in MUDT and in umbilical cord blood transplantation (UCBT) [91, 92]. In TCD haplo-SCT settings, Ciurea et al. [82] reported that three of four patients (75 %) that tested positive for pretransplant DSA (mean fluorescence intensity, MFI > 1500) failed to engraft, compared to 1 out of 20 patients (5 %) that tested DSA negative (*P* = 0.008), among 24 consecutive patients. In a study of 296 candidates for unmanipulated haplo-SCT with PT/Cy, the overall incidence of DSA was 15 %. Gladstone et al. [93] also found that DSA was associated with an increased risk of graft failure after transplantation. More recently, Chang et al. [12] reported that DSAs (MFI ≥ 10,000) were correlated to primary graft rejection (GR, *P* < 0.001) and that DSAs (MFI ≥ 2000) were strongly associated with poor graft function (PGF) in patients that received unmanipulated haplo-SCT with ATG. They also showed that primary GF, including GR and PGF, was associated with a significant increase in the incidence of TRM and with reduced DFS and OS [12, 20]. For patients with DSA, it is necessary to select a different donor. However, there is no generally accepted cutoff value for the mean fluorescence intensity of DSA in unmanipulated haplo-SCT with ATG [1, 52–55] or with PT/Cy [1, 36, 58, 59]. Overall, the association between DSA and graft failure was confirmed, both in TCD and in TCR haplo-SCT settings [12, 81, 82]. When a patient is positive for DSA (for example, DSA MFI ≥ 2000 in the Peking University Institute of Hematology), but the donor cannot be changed, a therapy must be given to target the DSA.

Currently in HSCT settings, desensitization methods have been applied, including plasma exchange, intravenous immunoglobulin, rituximab, and bortezomib [94, 95]. However, the efficacy of these strategies remains uncertain, due to the overall small number of patients treated and to the overall poor understanding of the mechanisms underlying DSA-mediated GF and PGF. Further elucidation of these mechanisms is essential to obtain critical insights into how desensitization approaches can be modified and what immuno-modifying therapies can be applied. That information will facilitate improvements in haplo-SCT outcomes.

In summary, DSA must be incorporated into the algorithm for haploidentical donor selection in unmanipulated haplo-SCT, with either the ATG or the PT/Cy modality. Therapies that target DSA might improve clinical outcomes for patients that are DSA positive and have only one haploidentical donor.

### Donor age

In haplo-SCT with TCD, no effects of donor age were observed on transplant outcomes. In unmanipulated haplo-SCT, Wang et al. [10] found that transplants from younger donors (age ≤30 years) showed less non-relapse mortality (NRM) and better survival than those from older donors. In previous studies, we found that a high dose of CD34^+^ cells in haplo-allografts could promote platelet engraftment, and that CD3^+^CD4^−^CD8^−^ T cells might contribute to a lower incidence of acute GVHD [96, 97]. More recently, researchers from Peking University also demonstrated that a young donor age (≤30 years) was associated with a higher count of CD34^+^ cells, CD3^+^CD4^−^CD8^−^ T cells, and monocytes in G-BM, G-PB, and mixed allografts of G-BM and G-PB [56]. The impact of donor age was also confirmed by researchers from Korea in unmanipulated haplo-SCT with ATG [98]. They found that donor age (>40 years) was associated with a higher incidence of grades II–IV acute GVHD. More recently, Jaiswal et al. [99] reported that age-related clonal hematopoiesis was commonly associated with increases in hematologic cancer risk and all-cause mortality. Those findings strongly argued for the benefit of selecting younger donors to minimize transfers of clonal hematopoiesis [100].

In summary, younger donors are preferred in unmanipulated haplo-SCT, with ATG or PT/Cy.

### Donor gender

For female donors, in general, age is correlated with parity. Older multiparous women may be the least-preferred donors for male recipients, due to the higher incidence of GVHD and the lower OS reported in some studies that focused on unrelated donor transplantations [101, 102]. Donor gender (female versus male) had adverse effects on the incidence of grades II–IV acute GVHD, both in unmanipulated haplo-SCT with PT/Cy and in TCR haplo-SCT with an ATG-based conditioning regimen [14, 36, 103]. Interestingly, in the largest study, the Peking University group showed that transplants from male donors were associated with significantly less NRM and better survival [10].

In summary, a male donor is preferred in unmanipulated haplo-SCT with ATG or with PT/Cy, due to the potential for superior survival.

### ABO compatibility

In both HLA-matched and HLA-mismatched settings, allogeneic SCT that involves a major ABO incompatibility requires mononuclear cell separation to prevent a hemolytic reaction. This procedure reduces the transplanted cell dose and may increase the likelihood of graft failure [104, 105]. When possible, transplant donors should not be selected when they have major ABO incompatibilities, to avoid graft manipulations that might reduce the nucleated cell dose, particularly the CD34^+^ cell dose [97]. Our experience at Peking University showed that a low number of CD34^+^ cells (less than 2.19 × 10^6^/kg) in the allograft was a critical factor associated with delayed platelet engraftment after unmanipulated haploidentical transplantation, in either adult or pediatric patients [97]. Those results suggested that, when no ABO-compatible donor was available, a donor with a minor ABO mismatch was preferable to a donor with a major mismatch, because the former was less likely to affect the number of hematopoietic stem cells infused. Thus, ensuring an adequate CD34^+^ cell dose in the allograft is the first step in promoting engraftment and decreasing the incidence of graft failure.

In summary, ABO compatibility should be considered when selecting the best donor in haplo-SCT with TCR; the order of selection should be ABO compatible, a minor ABO mismatch, and a major ABO mismatch.

### Killer immunoglobulin-like receptor mismatches and NK cell alloreactivity

#### Biology of NK cells

NK cells play a central role in viral immunity and tumor immune surveillance. The activity of NK cells is regulated by a balance between activating and inhibiting killer immunoglobulin-like receptors (KIRs) [106]. KIRs are inherited as one of two basic KIR haplotypes, termed group A and group B. Group A haplotypes have a fixed number of genes that encode inhibitory receptors (with the exception of the activating receptor, KIR2DS4). Group B haplotypes have a variable number of genes, including additional activating receptor genes [84, 85].

Because KIR and HLA class I genes segregate to different chromosomes, a tolerance mechanism is required to prohibit the development of autoreactive NK cells. Only NK cells that express inhibitory receptors for self-HLA class I can acquire full functional competence, a process referred to as “education” or “licensing” [23]. In contrast, potentially autoreactive NK cells remain in a hyporesponsive state. Thus, NK cells that are “licensed” or “educated” (highly responsive to non-self cells) express inhibitory KIRs that specifically recognize self-HLA ligands [107, 108]. Examples of inhibitory KIRs include the well-defined KIR2DL2/3, specific for the HLA-Cw group-1 epitope; KIR2DL1, specific for the HLA-Cw group-2 epitope, and KIR3DL1, specific for the HLA-Bw4 epitope [84]. Thus, when educated NK cells confront an allogeneic target, their KIR does not recognize the allogeneic HLA as an inhibitory self-HLA ligand; the lack of the inhibitory ligand mediates NK “alloreactivity” (they attack cells that lack self-recognition molecules) [109–112]. In fact, alloreactive NK cells must only express KIRs that do not engage with any HLA class 1 molecules present on allogeneic target cells. Moreover, for effective alloreactivity, NK cells must also lack expression of CD94/NKG2A, because its inhibitory ligand, HLA-E, is present on all HLA class I-positive cells [87, 113, 114].

#### Role of NK cell alloreactivity in haplo-SCT

Ruggeri et al. [110] showed that alloreactive NK cells in a mouse model provided the following benefits: (1) elimination of recipient acute myeloid leukemia (AML) cells; (2) destruction of recipient T cells, which permitted a conditioning regimen with reduced toxicity; and (3) ablation of recipient dendritic cells that trigger GVHD, which protected the recipient from GVHD. In this study, the authors also found that increased NK cell alloreactivity in humans, based on the “missing self” model, was associated with a decreased rate of relapse and improved survival in patients with AML but not in patients with ALL. However, Symons et al. [115] failed to demonstrate a positive effect of alloreactive NK cells in patients that received haplo-SCT with PT/Cy. In contrast, Huang et al. [11] showed that a high relapse rate following haplo-SCT was associated with missing self molecules or missing ligands in the hosts.

The discordant results mentioned above may reflect differences in NK functional recovery, determined by the licensing process under different haplo-SCT settings, and/or differences in the presence of T cells in the stem cell graft. More interestingly, the researchers in Huang’s group demonstrated that the host MHC class I could determine NK cell responses, following unmanipulated haplo-SCT with ATG [11, 23]. The functional recovery of donor-derived NK cells was higher in recipients that expressed ligands for donor inhibitory KIRs, and high functional NK recovery correlated with better relapse control. Those results were consistent with previous studies, which suggested that T cells may influence NK cell function via presentation of MHC. Although it remains to be determined by what mechanism(s) the presence of T cells in the allograft influence NK cell licensing, they appear to be clinically relevant. Moreover, NK licensing was observed to have extremely relevant clinical implications, such as relapse and survival.

Symons et al. [115] showed that, in haplo-SCT with PT/Cy, patients with the KIR AA haplotype exhibited significantly higher OS and EFS, when the donor had a KIR Bx haplotype (mismatched) rather than the KIR AA haplotype (matched). In haplo-SCT with negative depletion of CD3/CD19 in allografts, the relapse incidence was significantly reduced in patients with a haplotype B donor, both in adults with hematological malignancies [116] and in children with ALL [117]. However, this phenomenon was not observed in the Perugia or the Peking University protocols, which suggested that the benefit of using donors with KIR B haplotypes was only observed with specific haplo-SCT modalities; however, the mechanisms underlying this phenomenon warrant further study.

In summary, NK cell alloreactivity and KIR haplotype should be considered when choosing the best donor. For patients that receive unmanipulated haplo-SCT with ATG, the best donor will have matching KIR expression. For patients that receive TCR haplo-SCT with PT/Cy, the best donor should have at least one KIR B haplotype.

### NIMA mismatching

When a donor and recipient share inheritance of the paternal HLA haplotype, they are said to be mismatched for non-inherited maternal HLA antigens or NIMA (For more details, please see Fig. 1 in [86]). When a donor and recipient share inheritance of the maternal HLA haplotype, they are mismatched for non-inherited paternal HLA antigens or NIPA [88, 90, 118, 119]. Cells from NIMA-mismatched donors are expected to be less immunogenic than cells from NIPA-mismatched donors, because the contact between the immune systems of the mother and child during pregnancy diminishes the immune response of the child against NIMA. Using a mouse model, Aoyama et al. [87] demonstrated that the tolerogenic NIMA effect could be partly dependent on CD4^+^CD25^+^ regulatory T cells (Tregs). In TCR haplo-SCT, Wang et al. [10] and other researchers [88, 90, 120] showed that patients that received transplants from a NIMA-mismatched donor had a significantly lower incidence of acute GVHD than those that received transplants from a NIPA-mismatched donor. The Peking University group also found that immune recovery of naive Tregs was more rapid when patients received allografts from NIMA-mismatched donors than when they received allografts from NIPA-mismatched donors (unpublished data). That finding suggested that naive Tregs may play an important role in the tolerogenic NIMA effect. In addition, Kanda et al. [121] showed that a substantial proportion of long-term survivors after NIMA-mismatched haplo-SCT could discontinue the administration of immunosuppressive agents, despite the frequent occurrence of moderate to severe chronic GVHD. However, further studies are warranted to compare late sequelae between haplo-SCTs performed with NIMA- or NIPA-mismatched donors in a large, multicenter, prospective cohort.

**Fig. 1:**
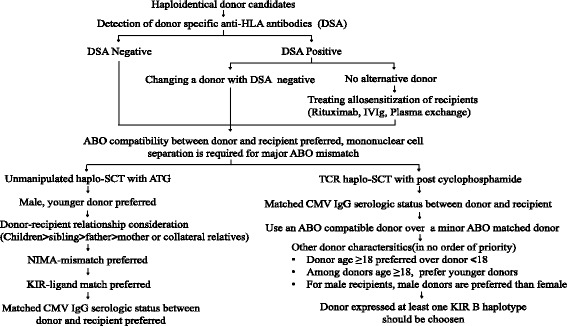
Algorithm for haploidentical donor selection in unmanipulated haplo-SCT with ATG and haplo-SCT with PT/Cy. Abbreviations: *haplo-SCT* haploidentical stem cell transplantation; *ATG* anti-thymocyte globulin; *PT/Cy* posttransplant cyclophosphamide; *TCR* T-cell replete; *IVIg* intravenous immunoglobulin; *CMV* cytomegalovirus; *NIMA* non-inherited maternal antigen; *KIR* inhibitory killer cell immunoglobulin-like receptor

Recently, Araki et al. [122] demonstrated that the number of cells that produced interferon-γ (IFN-γ) was significantly lower in a NIMA-exposed tolerance group than in a sensitization group, according to an MLR-ELISPOT assay in a murine model. That study raised the possibility that assays for measuring IFN-γ production in response to NIMA might be used clinically to predict the benefit of using NIMA-mismatched donors.

In summary, NIMA mismatching should be incorporated into the algorithm for selecting donors in unmanipulated haplo-SCT with ATG. The order of donor eligibility is first, NIMA mismatches, and second, NIPA mismatches.

## Family relationship or type of donor

Given the fact that parents, children, siblings, and collateral relatives are all potential haploidentical donors [9, 16, 19, 22], the effects of these variables on clinical outcomes were investigated by several groups [1, 10, 70]. In unmanipulated haplo-SCT with ATG, results from Huang et al.’s group in Beijing demonstrated that transplants donated by fathers were associated with less NRM, less acute GVHD, and better survival compared to those donated by mothers [10]. Transplants donated by children were associated with less acute GVHD than those donated by siblings. Transplants donated by older sisters were inferior to those donated by fathers, with regard to NRM and survival. Moreover, transplants donated by mothers were associated with significantly more acute and chronic GVHD and TRM than NIMA-mismatched, but not NIPA-mismatched, transplants donated by siblings [10]. However, Stern et al. [70] observed a survival advantage in patients with ALL or AML that received TCD-allografts from haploidentical maternal donors. The above-mentioned opposite results in the two studies may be related to differences in the conditioning regimens, GVHD prophylaxis, and allografts between the two groups [10, 70]. Zhang et al. [60] found that, when haplo-SCT was performed with collateral-related haploidentical donors (CRDs) or with immediate-related donors (IRDs), the 3-year probability of OS and LFS was similar, but the 2-year incidence of extensive chronic GVHD was significantly higher with CRDs than with IRDs (36.7 % versus 20.2 %, *P* = 0.03) [60]. The effects of donor-recipient relationships (parents or siblings) on TRM and LFS were also confirmed in patients with AML that received haplo-SCT with TCD [69].

In summary, the family relationship of a donor should be incorporated in the algorithm for selecting the best donor in unmanipulated haplo-SCT with ATG. The order of donor eligibility among relatives should be child, younger brother, older sister, father, mother, and a collateral relative [10].

## Donor and recipient serum CMV status

The effects of donor and recipient serum CMV status on clinical outcomes were demonstrated in HLA-matched transplantation settings [123]. Considering the effects of CMV status on outcomes [19, 69, 123], a group from Johns Hopkins [124] suggested that donors should have a CMV IgG serologic status similar to that of recipients. However, Wang et al. [9, 10] found that donor-recipient CMV serostatus matching was not associated with transplant outcomes. This discrepancy may be related to the higher incidence of CMV infections in Chinese compared to Western populations. Therefore, the effects of donor and recipient CMV status on haplo-SCT outcomes should be evaluated in a prospective, multicenter study.

In summary, donor and recipient CMV serostatus should be considered when choosing the best donor in unmanipulated haplo-SCT, particularly when patients receive haplo-SCT with PT/Cy; however, further study is needed to confirm the findings.

## Recommendations

Presently, a number of donor-related factors have been identified that affect haplo-SCT outcomes. These factors should be considered when selecting the best donor. Here, we have listed some expert opinions, based on available data from original literature:

- HLA matching: The effects of HLA disparity on transplantation outcomes has vanished, due to the improved approaches of unmanipulated haplo-SCT with ATG and haplo-SCT with PT/Cy.
- Donor-specific antibodies: DSA must be incorporated in the algorithm for haploidentical donor selection, both in unmanipulated haplo-SCT with ATG and in haplo-SCT with PT/Cy. Procedures to reduce DSA prior to transplantation should be considered for patients that have DSA against potential haploidentical donors.
- ABO compatibility: ABO compatibility should be considered in both unmanipulated haplo-SCT with ATG and haplo-SCT with PT/Cy.
- Serum CMV status: Among haploidentical donors, donor and recipient CMV serostatus should be considered, in both unmanipulated haplo-SCT with ATG and haplo-SCT with PT/Cy.
- Donor age: Among haploidentical donors, young males should be considered optimal, in both unmanipulated haplo-SCT with ATG and haplo-SCT with PT/Cy.
- Family relationship: Family relationships should be considered in unmanipulated haplo-SCT with ATG-based conditioning, with the following order of donor preference: child, younger brother, older sister or father, older sibling, mother, and collateral relatives.
- NIMA mismatches: NIMA mismatching should be incorporated into the algorithm for donor selection in unmanipulated haplo-SCT with the ATG protocol. The order of donor eligibility should be NIMA mismatches, followed by NIPA mismatches.
- NK cell alloreactivity: NK cell alloreactivity should be considered in choosing a donor for both unmanipulated haplo-SCT with ATG and haplo-SCT with PT/Cy.

According to these recommendations, we have proposed an algorithm for haploidentical donor selection (Fig. 1). When choosing the best haploidentical donor, one should keep the following caveats in mind. First, there is not a generally accepted haplo-SCT protocol that can be used in all transplant centers; therefore, a single variable (such as NK alloreactivity) may have different effects on clinical outcomes in patients that receive different haplo-SCT protocols [10, 19, 34, 36, 59, 70, 72, 77, 100, 125]. Second, with improvements in haplo-SCT modalities, the impact of some variables (such as HLA-locus mismatches) on transplant outcomes may vanish. Third, with increasing numbers of haplo-SCT cases, and with updated analyses of donor-related variables associated with transplant outcomes, some new factors may emerge [126, 127].

## Conclusions

Presently, TCR haplo-SCT modalities, particularly unmanipulated haplo-SCT with ATG or haplo-SCT with PT/Cy, have been widely accepted as a viable alternative for patients with no HLA-identical donor [1, 5, 6, 9, 10, 34–36, 72, 77, 128, 129]. Despite the challenges in promoting hematopoietic engraftment, in enhancing GVL effects, and in the lack of one universal haplo-SCT modality for most transplant centers, current evidence has indicated that selecting a best donor can improve transplant outcomes [10, 36, 59, 77, 81, 82, 115, 116, 130]. Therefore, employment of the currently available factors, including DSA, donor age, KIR-ligand mismatching, and NIMA mismatching, for guiding treatment is an accepted option in most centers [10, 36, 59, 77, 82, 100, 115, 116, 130]. Many recent excellent studies have advocated that donor selection should be incorporated into clinical trials. Although much work remains to be done, such as who is the best donor in subgroup patients (for example, high-risk AML), we believe that the best donor should be selected according to currently available knowledge, in combination with individualized conditioning regimens [46], optimal allografts [57], and stratification-directed GVHD prophylaxis, relapse prophylaxis, and treatment [131, 132]. This selection strategy will improve transplant outcomes, both in unmanipulated haplo-SCT with ATG and haplo-SCT with PT/Cy.
